# Education Convention of Kentucky

**Published:** 1833

**Authors:** 


### Art. XV. Education Convention of Kentucky.

It affords us great pleasure to say, that the Education Convention of the State of Kentucky, announced, in our last number, was well attended. On the invitation of the Rev. Mr. Peers, acting President of Transylvania University, we had the pleasure, with several distinguished gentlemen of Ohio and other States, of sitting in the Convention as an honorary member; and marked the progress of its deliberations with much gratification. Many of its members were gentlemen of talents and general influence; among whom we noted not a few of our own profession. The Convention adjourned, to meet in Frankfort on the 9th inst., when it is expected that a still larger number will attend, and that a variety of measures will be commenced, which must, in their consummation, improve the schools, academies and colleges, of that respectable and prosperous State.

All this is as it should be; and it is meet and proper, that the oldest member of the western sisterhood should be the first to hold a State Convention for the improvement of her institutions of learning, and the elevation of her literary character. The beneficial effects of such improvement, will speedily manifest themselves in various ways, but in none more strikingly than the amelioration of the condition of the medical profession of that State, already highly respectable in every thing but its literature, which even now is not inferior to any other State in the west.

